# Use of Spineless Cactus (*Opuntia ficus indica f. inermis*) for Dairy Goats and Growing Kids: Impacts on Milk Production, Kid's Growth, and Meat Quality

**DOI:** 10.1100/2012/321567

**Published:** 2012-03-12

**Authors:** M. Mahouachi, N. Atti, H. Hajji

**Affiliations:** ^1^Ecole Supérieure d'Agriculture du Kef, Le Kef 7119, Tunisia; ^2^INRA-Tunisie, Laboratoire des Productions Animales et Fourragères, Rue Hédi Karray, Ariana 2080, Tunisia; ^3^Institut National Agronomique de Tunis, Tunis 2019, Tunisia

## Abstract

The objective of this study was to determine the effect of spineless cactus incorporation in food of dairy goats and growing kids on milk production and composition and on kid's growth and meat characteristics. Two experiments were conducted on Tunisian local goats. In the first, 30 females were divided into two groups; goats of Control group were reared on grazing pasture receiving indoor 0.5 kg of hay and 0.4 kg of concentrate. Goats for the second group (Cac-FL) were kept in feedlot and fed cactus ad libitum more 0.5 kg of hay and 0.4 kg of concentrate. In the second experiment, 14 kids were divided into 2 groups receiving 600 g of hay. The Control group received ad libitum a concentrate containing 130 g crude protein (CP) per kg of dry matter. The second group received cactus ad-libitum plus the half concentrate quantity of control one with 260 g CP/kg DM (Cactus). The daily milk production averaged 485 ml for Control group and 407 ml for Cac-FL one. The milk fat content was significantly higher for Control than Cac-FL group. In the second experiment, animals in Control and Cactus groups had similar growth rate. Carcass fat was significantly lower in Cactus than in the Control group. Cactus in the diet was associated with more C18:2 and conjugated linoleic acid as well as a higher proportion of PUFA than Control ones.

## 1. Introduction

The arid and semiarid areas are characterized by limited food resources, and the production of green fodder is rare, particularly during the hot and dry season (summer) when the animals are strongly complemented by food concentrate [1]. To face these critical periods, the search for other natural resources is needed to better sustain the dairy production and the growth of the young animals and to preserve breeding continuity. In some arid regions, the spineless cactus or prickly peartree (*Opuntia ficus indica f. inermis*), which is useful for ground conservation and reduction of streaming, is widely cultivated and could be used as green fodder in all seasons. The digestibility of the ration and the growth of the lambs nourished with cactus were studied [2] whereas works on meat quality or dairy ewe and goat fed cactus are rare [3]. The objective of this work is to study the production and the composition of the nursing goat's milk eating cactus in comparison with the production on pasture of the natural range and its effects on kid's growth and meat characteristics.

## 2. Material and Methods

### 2.1. Animals and Feeding

Two experiments were conducted on Tunisian local goats (Maure breed). In the first experiment, 30 adult goats were divided into two groups according to the parity, the milk production with the first control, and the number of nursed kids. Goats for Control group were reared on grazing pasture during 6 hours per day and received indoor per head and day 0.5 kg of oat hay and 0.4 kg of concentrate (180 g crude protein (CP) per kg dry matter (DM)). Goats for the second group (Cac-FL) were kept in feedlot and fed spineless cactus ad libitum plus 0.5 kg of the same hay and 0.4 kg of the same concentrate. In the second experiment, 14 kids (17.4 kg live weight) and two kinds of concentrates were used. The concentrates C130 and C260 contained 130 and 260 g CP/kg DM, respectively. Animals were divided into 2 groups receiving 600 g of oat hay. The first group (Control) received 600 g of C130 concentrate, while the second group (Cactus) received cactus ad libitum plus 300 g of C260 concentrate. Animals were allowed 84 days in this trial and then were slaughtered.

### 2.2. Animal Performance Recording and Sampling

For the first experiment, the goats and kids were weighed weekly before eating. Dairy control was bi monthly. For this control and during the suckling phase, the kids were separated from their mothers during 8 hours; the goats were then milked and the quantity of milk obtained was multiplied by 3 to have the total daily milk production. For each control, an individual milk sample was reserved for the chemical analysis. For the second experiment, kids were weighed weekly before eating. After slaughter, carcasses were weighed then split into two halves. The left sides were separated into five joints, which were dissected into fat, muscles, and bones. Samples of the *longissimus dorsi* muscles were taken for chemical meat analysis.

### 2.3. Chemical Analysis

The individual milk samples were kept (4°C) and analyzed for milk fat (MF) and protein (MP) using a MilkoScan 4000 (FOSS ELECTRIC, Integrated Milk Testing). For chemical composition of the Muscle *longissimus dorsi*, DM was determined by lyophilisation, mineral content by ashing at 600°C for 8 h and Nitrogen by Kjeldahl method (CP = *N* × 6.25). Meat lipids were calculated as DM minus the sum of protein and ash. The fatty acid composition of the intramuscular (i.m.) fat was analyzed after extraction and methylation with K-OH on chromatograph (HP-5890) equipped with a flame ionization detector and split (1 : 24) injector.

### 2.4. Statistical Analysis

Statistical analysis was performed by analysis of variance using the GLM procedure of SAS (1989). The effect of dietary treatment was analyzed on milk production and composition for the first experiment and on kid growth and carcass and meat quality for the second.

## 3. Results and Discussion

### 3.1. First Experiment

#### 3.1.1. Milk Production

Daily milk production was not significantly affected by diet treatment (Table 1). The peak of lactation was observed towards the 4th week for Ca-FL group and the 6th week for the Control group. The increase in milk production was associated with a decrease in goats' weight underlining the mobilization of the body reserves. Then the production level dropped with a persistence coefficient of 0.85. The milk production level remained relatively low; the limited concentrate supply (maximum 400 g) with cactus or pasture in dry year may be the reason of this weak production, since the performances of the same herd are higher (600 to 800 ml/j) with other diets [1].

#### 3.1.2. Chemical Milk Composition

The protein content had the same value during all the experimental period without significant difference between the experimental groups (Table 1). The evolution of the milk fat shows more fluctuations than that of protein. The milk fat rate was higher for pasture group than Cac-FL (Table 1), particularly at the end of the lactation. The variability of the grazed species would result in ingestion of plants rich in the most widespread fatty acids precursor in the goat's milk. The fat content increased considerably at the end of the lactation which corresponds to the period of milk production decrease confirming the negative correlation between the dairy production and the fat content rate [4, 5]. 

### 3.2. Second Experiment

#### 3.2.1. Kid's Growth

The use of cactus, despite the reduction of concentrate, tends to improve the ADG from 35 g/day (Control) to 55 g/day (Cactus); this result is in relationship with the high energy and protein intake of groups fed cactus diets, which characterized by a high DM intake and digestibility. These results confirmed partially those of Negesse et al. [6] and Titi et al. [7] who observed a linear increase in weight gain for Saanen kids and Black goat kids, fed with higher levels of diet CP. These results showed clearly that cactus, largely used in arid and semiarid areas for rangeland rehabilitation, is also a potential cost-effective fodder for weaned kids in semiarid areas. However, the slight difference in growth rates among treatments would suggest that the nutritive value of diets was not the only limiting factor but reflects also the low potential growth rate of this local breed.

#### 3.2.2. Carcass and Meat Composition

The results concerning slaughter BW, carcass weight, and importance of fat and muscle tissues were presented in Table 2. The muscle weight was similar for both groups. For this similar amount of muscle, the fat amount was 150% for Control group compared to Cactus one (*P* < 0.001). As proportions of carcass weight, there were significant effects (*P* < 0.001) of diet on muscle and fat values, carcasses of kids given cactus diet presented relatively less fat (105 g/kg) and more muscle (62 g/kg) than those fed control diet (135 g/kg fat and 58 g/kg muscle). It is necessary to point out that cactus supply decreased the fat tissue weight and proportion and increased muscle proportion in the carcass suggesting that carcasses of fed cactus goats were leaner than carcasses produced on grain diets, which confirmed other results [3]. Hence, cactus acts on carcass composition as a green forage confirming other results where animals (steers, beef and lambs) raised on pasture displayed a greater proportion of muscle and a lower percentage of fat compared to concentrate fed animals [8–10].

Results of chemical characteristics determined on LD muscles were shown in Table 2. No significant differences were found for moisture, crude protein, and ash contents between treatment groups. The crude fat decreased from 151 to 91 g/kg DM for Control and Cactus groups, respectively. So, meat of kids fed on cactus was leaner than that of Control.

#### 3.2.3. Fatty Acid Composition

Results obtained for proportions of i.m. fatty acid composition for both groups (Table 3) were in concordance with Banskalieva et al. [11] for other goat breeds, showing the prevalence of oleic (C18:1), palmitic (C16:0), stearic (C18:0), and linoleic (C18:2) FA, which accounted for about 910 g/kg of total fatty acids. However, CLA and DPA (C22:5n-3), healthy FA, were significantly higher (*P* < 0.001) in the Cactus group than Control one (Table 3). These results are in accordance with other reports, which compared fatty acid composition of grass-fed to concentrate-fed lambs [12], steers or cattle [10, 13], and goats [3]. There was no significant influence of feeding (*P* > 0.05) on the SFA, PUFA, and the PUFA/SFA ratio.

## 4. Conclusions

The cactus used as basic food, in addition to moderate quantity of concentrate, could generate a dairy production of the goats comparable or slightly lower than those of the animals led on rangeland in average years. Moreover, the surface reserved for the production of cactus represents only the tenth of that reserved for the pasture, which makes cactus a solution for the stockbreeders who do not have sufficient rangeland. 

Kids reared by cactus feeding produce meat with enhanced nutritional quality for the consumer due to lower carcass adiposity, lower intramuscular fat content, and a higher accumulation of beneficial fatty acids, n-3 FA, and CLA.

Given the increasing cost of concentrates, raising kids under cactus regimen could be more economic than the conventional regimen.

## Figures and Tables

**Table 1:** Milk production and composition.

Parameter	Pasture	Cac-FL	stat
Daily milk production (mL)	485 ± 42	403 ± 43	ns
Fat content (%)	3.9 ± 0.17	3.7 ± 0.17	*
Protein content (%)	2.8 ± 0.09	2.8 ± 0.09	ns

*P < 0.05; ns: not significant (*P* < 0.05).

**Table 2:** Slaughter BW, carcass weight, and importance of fat and muscle tissues for kids receiving cactus or conventional diets.

	Control	Cactus	stat
SBW, kg	21.2	21.4	ns
Cold carcass weight, kg	9.1	9.4	ns
Muscle, g	5109	5114	ns
Muscle, g/kg	570^a^	622^b^	***
Fat, g	1275^a^	810^b^	***
Fat, g/kg	142^a^	101^b^	***
Protein, g/kg DM	800	858	ns
Fat, g/kg DM	151	91	*

***P < 0.001; *P < 0.05; ns: not significant (*P* > 0.05).

**Table 3:** Average fatty acid (FA) profile in meat fat of kids, g/kg.

Measurement	C130	C260-Cac	*P*
C16	188	196	ns
C16:1	27.0	23.5	ns
C18	140	175	ns
C18:1	547	504	ns
C18:2n-6	22.8	23.8	ns
C18:3n-3	1.5	1.8	ns
CLA (cis-9, trans-11)	1.5^A^	2.2^A^	***
C20:4	8.1	10.2	ns
EPA (C20:5n-3)	0.3	0.9	*
DPA (C22:5n-3)	0.17^A^	1.7^B^	***
SFA	366	400	ns
MUFA	588	539	ns
PUFA	37.2	44.4	ns

SFA: sum of saturated fatty acids: C14:0 + C15:0 + C16:0 + C17:0 + C18:0.

MUFA: sum of monounsaturated fatty acids: C14:1 + C16:1 + C17:1 + C18:1 + C20:1.

PUFA: sum of polyunsaturated fatty acids: C18:2 + C18:3 + CLA + C20:2 + C20:3 + C20:4 + EPA + DP.

***P < 0.001; *P < 0.05; ns: not significant (*P* > 0.05).
